# Association between Provider Volume and Healthcare Expenditures of Patients with Oral Cancer in Taiwan: A Population-Based Study

**DOI:** 10.1371/journal.pone.0065077

**Published:** 2013-06-04

**Authors:** Li-Fu Chen, Hsu-Chueh Ho, Yu-Chieh Su, Moon-Sing Lee, Shih-Kai Hung, Pesus Chou, Ching-Chieh J. Lee, Li-Chu Lin, Ching-Chih Lee

**Affiliations:** 1 Department of Emergency, National Yang-Ming University Hospital, I-Lan, Taiwan; 2 Department of Otolaryngology, Buddhist Dalin Tzu Chi General Hospital, Chiayi, Taiwan; 3 Division of Hematology-Oncology, Department of Internal Medicine, Buddhist Dalin Tzu Chi General Hospital, Chiayi, Taiwan; 4 Department of Radiation Oncology, Buddhist Dalin Tzu Chi General Hospital, Chiayi, Taiwan; 5 Community Medicine Research Center and Institute of Public Health, National Yang-Ming University, Taipei, Taiwan; 6 School of Medicine, Tzu Chi University, Hualian, Taiwan; 7 Department of Occupational Medicine, Municipal Hsiao-Kang Hospital, Kaohsiung, Taiwan; 8 Department of Medical Research, Buddhist Dalin Tzu Chi General Hospital, Chiayi, Taiwan; 9 Graduate Institute of Elder Education, National Chung Cheng University, Chiayi, Taiwan; Boston Children’s Hospital, United States of America

## Abstract

**Background:**

Oral cancer requires considerable utilization of healthcare services. Wide resection of the tumor and reconstruction with free flap are widely used. Due to high recurrence rate, close follow-up is mandatory. This study was conducted to explore the relationship between the healthcare expenditure of oncological surgery and one-year follow up and provider volume.

**Methods:**

From the National Health Insurance Research Database published by the Taiwanese National Health Research Institute, the authors selected a total of 1300 oral cancer patients who underwent tumor resection and free flap reconstruction in 2008. Hierarchical linear regression analysis was subsequently performed to explore the relationship between provider volume and expenditures of oncological surgery and one-year follow-up period. Emergency department (ED) visits and 30-day readmission rates were also analyzed.

**Results:**

The mean expenditure for oncological surgery was $11080±4645 (all costs are given in U.S. dollars) and $10129±9248 for one-year follow up. For oncological surgery expenditure, oral cancer patients treated by low-volume surgeons had an additional $845 than those in high-volume surgeons in mixed model. For one-year follow-up expenditure, patients in low-volume hospitals had an additional $3439 than those in high-volume hospitals; patient in low-volume surgeons and medium-volume surgeons incurred an additional expenditure of $2065 and $1811 than those in high-volume surgeons. Oral cancer patients treated in low-volume hospitals incurred higher risk of 30-day readmission rate (odds ratio, 6.6; 95% confidence interval, 1.6–27).

**Conclusions:**

After adjusting for physician, hospital, and patient characteristics, low-volume provider performing wide excision with reconstructive surgery in oral cancer patients incurred significantly higher expenditure for oncological surgery and one-year healthcare per patient than did others with higher volumes. Treatment strategies adapted by high-volume providers should be further analyzed.

## Introduction

Oral cancer is among the 10 most common forms of cancer [1]. A trend of rising incidence has been noted on a global scale in Western countries as well as Asian countries such as Taiwan [2], [3]. Of all cancers in Taiwanese males, oral cancer has been ranked fourth in incidence and mortality since 1995. Furthermore, an increasing number of young patients with oral cancer has also been observed [4]. The increasing economic burden of oral cancer treatment is an obvious consequence [5], [6]. The main treatment modality of oral cancer is wide resection of the tumor and reconstruction with or without adjuvant chemo-radiotherapy. With advances in the resection of tumors and flap reconstruction, economic and functional outcomes have become more important [2], [4].

The fact that increased case load is associated with improved outcomes has been noted for three decades in many areas of health care, including acute myocardial infarction, many types of high-risk operations and cancer surgery [7], [8], [9], [10], [11]. This phenomenon could be partly explained by the understanding “practice makes perfect”; “selective referral” may be an alternative explanation in other cases [12], [13]. Previous review had revealed that a significant volume effect was evident for the majority of gastrointestinal cancer; however, such as positive volume-outcome relationship is not well validated for other procedures [14].

At present, there is little information on resection and reconstruction and follow-up expenditure for oral cancer [15]. The purpose of this study was to examine the relationship between provider volume and expenditure of for oncological surgery and one-year follow-up period in oral cancer patients within a population-based database. The association of the readmissions to the hospitals and emergency department visits and the provider caseload were also explored.

## Materials and Methods

### Ethics Statement

This was a retrospective analysis of hospital and ambulatory visit administrative data provided by the National Health Research Institute for all patients with newly diagnosed oral cancer in 2008. This study was initiated after approval by the Institutional Review Board of Buddhist Dalin Tzu Chi General Hospital, Taiwan. Since all identifying personal information was stripped from the secondary files before analysis, the review board waived the requirement for written informed consent from the patients involved.

### Study Database

We used data from 2008 to 2009 from the National Health Insurance Research Database (NHIRD), which covered medical benefit claims for over 23 million people in Taiwan (approximately 99 percent of the island’s population). The database contains a registry of contracted medical facilities, a registry of board-certified physicians, and monthly summaries of all inpatient and ambulatory visits claims. All oral cancer patients (International Classification of Disease, Ninth Revision, Clinical Modification codes 140, 141, 143 and 145 who had received wide excision and free-flap operation (procedure 62032B to 62038B) in 2008 were included. In total, there were 1300 patients with oral cancer who underwent wide excision with free-flap reconstruction performed by 196 surgeons and 37 hospitals in 2008.

Surgeons were sorted by their total patient volume using unique surgeon identifiers in this database. The 1300 patients were sorted into three approximately equal groups based on surgeon volume: ≦8 cases (low), 9–21 cases (medium), and 22–96 cases (high) (Appendix S1) [16], [17], [18]. Hospitals were sorted using similar methods, and the caseloads were as 1–39 cases (low), 40–76 cases (medium) and 86–244 cases (high).

### Statistical Analysis

The key dependent variable of interest was the expenditure of each hospital admission for oncological surgery (wide resection, neck dissection, and free-flap reconstruction), and one-year follow-up expenditure. The expenditure included actual resources utilization, and charges which were paid by the patients and Bureau of National Health Insurance in Taiwan. The key independent variables were the oral cancer resection with reconstruction volume groups (low, medium, or high) for the surgeons and hospitals. Patient characteristics included age, gender, individual socioeconomic status (SES), urbanization and region of patients’ residence and medical illness. The recoding and definition of SES and urbanization of residence was mentioned in our previous study [19]. The medical illness of each patient was based on the Charlson Comorbidity Index score, which is widely used for risk adjustment in administrative claims data sets. We used a modified Charlson Comorbidity Index score, which is calculated as the sum of weighted scores based on the relative mortality risk for 19 conditions [20]. Surgeons’ age and teaching level of hospital were also recorded.

We further reported the visits to the emergency department (ED) and readmission to the hospital within 30 days after discharge from oncological surgery as the outcomes of care between different caseload groups in order to confirm whether lower costs may lead to higher ED visits or readmission rate in the follow-up period [21].

The SPSS version 15 (SPSS Inc., Chicago, IL) was used to analyze this data. The hierarchical linear regression model was used to analyze the relationship between the main outcomes of the different caseload groups and those of the reference group after adjusting for hospital, surgeon characteristics and patient demographics. In this study, the hierarchical linear regression method was used because of concern for the potential clustering effect in a hospital. A hospital-level random effect might account for possible correlations between hospitalization costs within a hospital’s panel simply because of hospital policies, procedures, or physician compensation mechanisms that were unique to that hospital. Multiple logistic regression model was performed to explore the association of provider volume and 30-day readmission rate and ED visits. A two-tailed value of p<0.05 was used to determine statistical significance.

## Results

Patient characteristics are detailed in Table 1. The mean age of oral cancer patients was 52±10 years. Oral cancer patients treated by low-volume surgeons were more likely to have low SES, resided in northern and southern/eastern Taiwan, and underwent treatment in regional hospitals, compared with those treated by high-volume surgeons. Oral cancer patients treated by low-volume hospitals were more likely to have moderate SES, resided in rural areas and southern/eastern Taiwan, had higher CCIS, and received treatment in regional hospitals, compared with those treated in high-volume hospitals.

**Table 1:** Baseline characteristics of oral cancer patients (n = 1300).

Characteristic	Surgeon caseload											Hospital caseload								
	High-volume (n = 436)					Medium-volume(n = 453)			Low-volume(n = 411)		Pvalue	High-volume(n = 436)			Medium- volume(n = 453)			Low- volume(n = 411)		Pvalue
	n				(%)	n	(%)		n	(%)		n	(%)		n		(%)	n	(%)	
Age, years (mean ±SD)	53±10					52±10			53±11		0.443	53±11			52±10			53±10		0.645
Gender											0.684									0.618
Male	429				(94)	399	(95)		407	(95)		412	(95)		429		(95)	394	(96)	
Female	26				(6)	19	(5)		20	(5)		24	(5)		24		(5)		(4)	
Individual SES											0.039									0.041
High	94				(21)	70	(17)		56	(13)		88	(20)		76		(17)	56	(14)	
Medium	187				(41)	194	(46)		196	(46)		175	(40)		199		(44)	203	(49)	
Low	174				(38)	154	(37)		175	(41)		173	(40)			178	(39)	152	(37)	
Urbanization of patients’ residence											0.784									0.047
Urban		87			(19)	91	(22)		92	(21)		86	(20)		101		(22)	83	(20)	
Suburban		208			(46)	182	(43)		196	(46)		219	(50)		198		(44)	169	(41)	
Rural		160			(35)	145	(35)		139	(33)		131	(30)		154		(34)	159	(39)	
Geographic region of patients’ residence											<0.001									0.018
Northern		138			(30)	139	(33)		183	(43)		156	(36)		159		(35)	145	(35)	
Central		168			(37)	98	(23)		91	(21)		119	(27)		145		(32)	93	(23)	
Southern/Eastern		149			(33)	181	(43)		153	(36)		161	(37)		149		(33)	173	(42)	
CCIS											0.116									<0.001
0		273			(60)	221	(53)		225	(53)		265	(61)		219		(48)	235	(57)	
1–6		158			(35)	163	(39)		171	(40)		151	(35)		187		(41)	154	(38)	
>6		24			(5)	34	(8)		31	(7)		20	(5)			47	(10)	22	(5)	
Teaching level of hospitals											<0.001									<0.001
Medical center			411	(90)		313	(75)		271	(64)		350	(80)		453		(100)	192	(47)	
Regional hospital			44	(10)		105	(25)		156	(36)		86	(20)		0		(0)	219	(53)	

CCIS, Charlson comorbidity index score; SES, Socioeconomic status.

Table 2 shows the surgeon and hospital characteristics. The high volume group surgeons were the oldest (P = 0.047). There was no statistical difference between the provider volume and the urbanization and areas of hospitals where they practiced.

**Table 2:** Surgeon and hospital characteristics.

Variable	Surgeon caseload					Hospital caseload			
	High-volume(22–96)		Medium-volume(9–21)	Low-volume(1–8)	Pvalue	High-volume(86–244)	Medium-volume(40–76)	Low-volume(1–39)	Pvalue
Total no. of surgeons/hospitals	12		30	154		3	8	26	
Age, years									
Mean ±SD	47±7		41±6	41±8	0.047				
Range	36–59		31–56	30–75					
Gender					0.372				
Male	12		30	147					
Female	0		0	7					
Urbanization of hospital location					0.148				0.090
Urban	8		12	50		2	4	5	
Suburban	3		16	83		0	4	18	
Rural	1		2	21		1	0	3	
Geographic region of hospital location					0.777				0.713
Northern	5		13	81		1	4	13	
Central	3		5	23		0	2	5	
Southern/Eastern		4	12	50		2	2	8	
Caseload (Mean ±SD)	38±20		14±4	3±2	<0.001	145±86	57±13	16±14	<0.001

SD, standard deviation.

**Figure 1:**
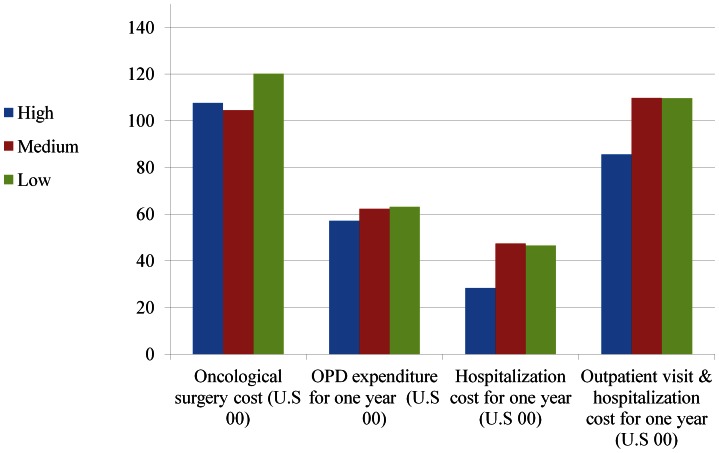
Expenditures of oral cancer patients for oncological surgery and one-year follow up in surgeons with different caseloads.

**Figure 2:**
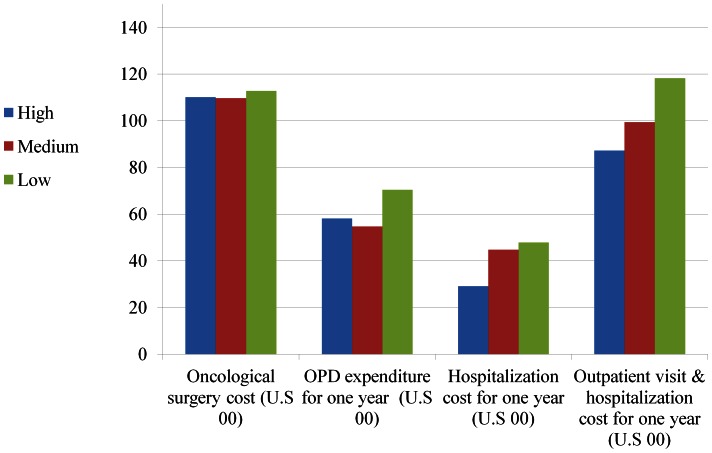
Expenditures of oral cancer patients for oncological surgery and one-year follow up in hospitals with different caseloads.

The mean expenditure for oncological surgery was $11080±4645 (all costs are given in U.S. dollars) and healthcare expenditure for 12-month follow-up period was $10129±9248 (Appendix S2). Figure 1 & 2 depict the unadjusted means of expenditure between the three caseload groups in surgeons and hospitals. The spending of oncological surgery and 12-month follow-up period for oral cancer patients treated in low-volume surgeons was $1248 and 2406 higher than the spending for those treated by high-volume surgeons (Table 3). Oral cancer patients treated in low-volume hospitals incurred higher one-year follow-up expenditure of $3107 than those in high-volume hospitals.

**Table 3:** Healthcare expenditure of oncological surgery and one-year follow-up period for oral cancer patients (n = 1300).

Characteristic	Surgeon caseload				Hospital caseload				
	High-volume	Medium-volume	Low-volume	Pvalue	High-volume	Medium- volume	Low-volume	Pvalue	
**Spending for oncological surgery**									
Oncological surgery cost(mean ±SD)	10769±3499	10460±4254	12017±5802	<0.001	11012±4612	10969±4081	11274±5231	0.586	
**Spending for 12-month follow-up period**									
OPD expenditure(mean ±SD)	5721±5264	6234±5014	6310±5806	0.207	5808±5405	5471±4963	7039±5653	<0.001	
Hospitalization cost(mean ±SD)	2840±5360	4742±8126	4658±7400	<0.001	2913±5382	4471±7330	4789±8143	<0.001	
OPD & hospitalizationexpenditure (mean ±SD)	8562±8046	10976±9830	10968±9653	<0.001	8721±8116	9941±9159	11828±10176	<0.001	

**Figure 3:**
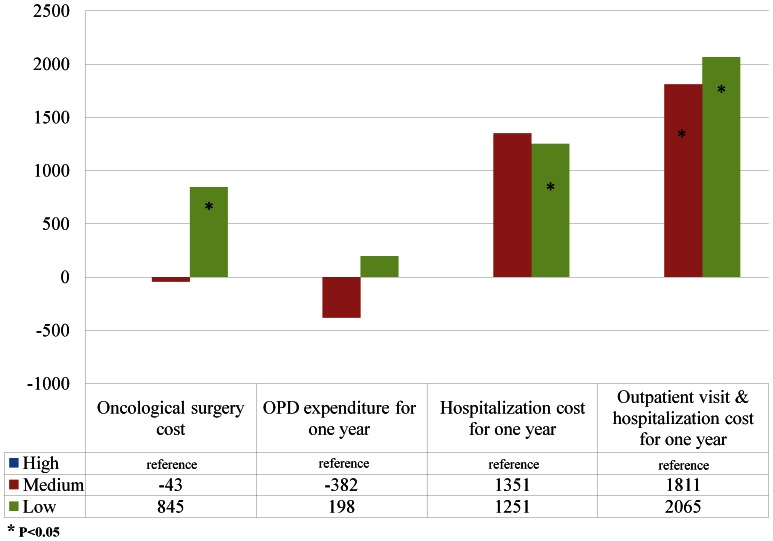
The difference of spending relative to the reference group (high-volume surgeons) in mixed models.

**Figure 4:**
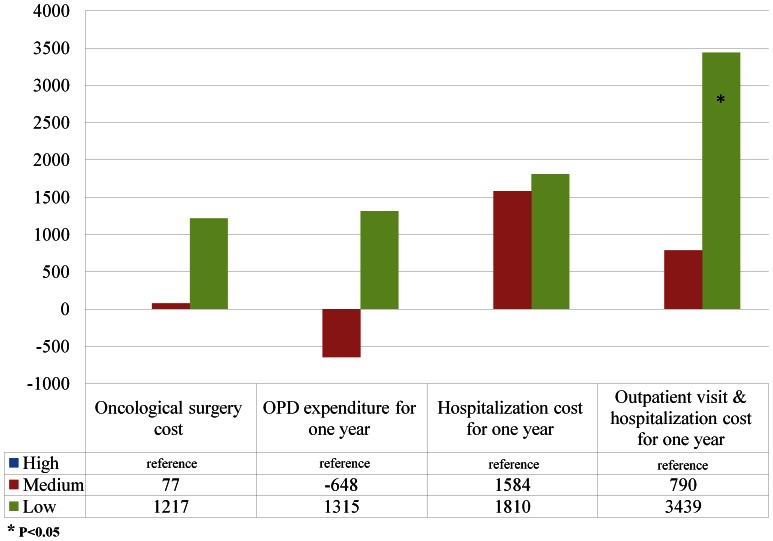
The difference of spending relative to the reference group (high-volume hospitals) in mixed models.

After adjusting for physician, hospital, and patient characteristics, the hierarchical linear regression revealed that the cost for oncological surgery per patient for low-volume surgeons was $845 higher than those of high-volume surgeons (P = 0.03), and $2065 higher than those of high-volume surgeons in healthcare expenditures for the 12-month follow-up period (P = 0.007) (Table 4 & Figure 3). Oral cancer patients treated in low-volume hospitals incurred higher one-year follow-up expenditure of $3439 than those in high-volume hospitals in mixed model (P = 0.004)(Table 4 & Figure 4).

**Table 4:** Summarized the mixed model results (n = 1300)*.

Characteristic	Surgeon caseload							Hospital caseload							
	High- volume	Medium-volume			Low-volume			High-volume	Medium-volume			Low-volume			
		β**	SE	Pvalue	β**	SE	Pvalue		β**	SE	Pvalue	β**	SE	Pvalue	
**Spending for oncological surgery**															
Oncological surgery cost	reference	−43	458	0.925	845	390	0.030	reference	79	1670	0.963	1217	1520	0.430	
**Spending for 12-month follow-up period**															
OPD expenditure	reference	−383	543	0.482	198	472	0.676	reference	−648	1085	0.557	1315	1002	0.204	
Hospitalization cost	reference	1350.91	701	0.055	1251	623	0.045	reference	1584	1311	0.250	1810	1217	0.160	
Outpatient & hospitalization cost	reference	1811	818	0.030	2065	763	0.007	reference	790	841	0.391	3439	836	0.004	

SE, standard error.

* Adjusted variables were the patients’ diagnosed age, gender, CCIS categories, urbanization and region of patients’ residence individual, socioeconomic status, surgeon’s age, and teaching level of hospitals.

** Parameter estimate.

Oral cancer patients treated in low-volume hospitals incurred higher rate of 30-day readmission event; however, oral cancer patients treated by low-volume surgeons had higher ED visits (Table 5). After adjusting other factors, oral cancer patients treated in low-volume hospitals incurred higher risk of 30-day readmission rate (odds ratio, 6.6; 95% confidence interval, 1.6–27) (Table 6).

**Table 5:** Emergency department visits and 30-day readmission rate for oral cancer patients (n = 1300).

Characteristic	Surgeon caseload							Hospital caseload							
	High-volume		Medium-volume		Low-volume		Pvalue	High-volume		Medium-volume		Low-volume		Pvalue	
	n	(%)	n	(%)	n	(%)		n	(%)	n	(%)	n	(%)		
Emergency department visits							0.026							0.773	
Yes	97	(21)	79	(19)	113	(27)		102	(23)	98	(22)	89	(22)		
No	358	(79)	339	(81)	314	(73)		334	(77)	355	(78)	322	(78)		
30-day readmission							0.188							0.012	
Yes	7	(2)	8	(2)	14	(3)		4	(1)	9	(2)	16	(4)		
No	448	(98)	410	(98)	413	(97)		432	(99)	444	(98)	395	(96)		

**Table 6:** Adjusted odds ratio of emergency department visits and 30-day readmission for oral cancer patients (n = 1300)*.

Characteristic	Emergency department visits			30-day readmission		
	Odds ratio	95% CI	P value	Odds ratio	95% CI	P value
Surgeon caseload						
High-volume	1			1		
Medium-volume	0.87	(0.57–1.31)	0.499	0.45	(0.12–1.63)	0.223
Low-volume	1.33	(0.91–1.93)	0.145	0.92	(0.27–3.16)	0.896
Hospital caseload						
High-volume	1			1		
Medium-volume	0.93	(0.65–1.33)	0.683	3.03	(0.86–10.64)	0.084
Low-volume	0.85	(0.56–1.29)	0.445	6.62	(1.60–27.36)	0.009

95% CI, 95% confidence interval.

* Adjusted variables were the patients’ diagnosed age, gender, CCIS categories, urbanization and region of patients’ residence, individual socioeconomic status, surgeon’s age, and teaching level of hospitals.

## Discussion

We found significantly higher adjusted expenditure for oncological surgery of oral cancer patients treated in low-volume surgeons and more healthcare expenditure for additional 12-month follow-up period treated by low-volume surgeons and low-volume hospitals. Low-volume surgeons had an additional 8% higher expenditure for oncological surgery and 20% higher expenditure for 12-month follow-up expenditures than high-volume surgeons after adjusting other factors; oral cancer patients treated by low-volume hospitals incurred an additional 34% higher spending for 12-month follow up than those in high-volume hospitals. Patients treated in low-volume hospitals incurred higher 30-day readmission rate. Given the high worldwide health care costs for oral cancer, our findings have significant implications for professional organizations and policy makers.

The strengths of our study are that it is based upon a large population (n = 1300), the population experienced almost complete follow-up of OPD visits and hospitalization (99%) and had routine monitoring of diagnosis accuracy by the National Health Insurance Bureau of Taiwan. Previous study explored the expenditure for oncological surgery alone in oral cancer [15]. Our series explored the association between both the surgeon and hospitals caseload and the spending for oncological surgery and one-year follow-up period. Our finding of an inverse volume-expenditure relationship is consistent with previous studies of the treatment of stroke, and percutaneous transluminal coronary angioplasty, which found lower costs per discharge for patients treated by high-volume groups [22], [23]. To provide insight whether efforts to control expenditure in oncological surgery may lead to higher complications in the follow-up period, our data further calculated the additional one-year follow-up expenditure, ED visits and 30-day readmission rate for oral cancer patients treated by providers with different caseload [21]. One-year follow-up expenditure remained higher in those treated in low-volume surgeons or hospitals. After adjusting other factors, oral cancer patients in low-volume hospitals had higher 30-day readmission rate.

Surgeons and hospitals caseload could affect the spending or complications through different mechanism. Gruen et al. stated that surgeon caseload may affect the outcomes through patient selection, preoperative and intraoperative decision making, and surgical techniques [14]. The “practice makes perfect” hypothesis suggests that larger case loads will help surgeons or hospital staff develop greater skills, implement the treatment process better, and be more cost effective [7], [12]. Resection of oral cancer, neck dissection, and reconstruction involve extremely tedious and lengthy surgery. High-volume surgeons with well-trained staff may perform surgery with a shortened operation period, appropriate antimicrobials and procedures, and well-designed discharge planning, therefore having fewer complications, shorter hospital stays and lower costs [8], [24], [25]. Considering selective referral effect, high-volume surgeons were less likely to admit oral cancer patients with low SES in our series; however, there is no definite data to support that high-volume surgeons treated oral cancers with earlier stage. For postoperative one-year follow-up expenditure, oral cancer patients in high-volume hospitals had lower spending. This is in agreement with previous study which postulated that high-volume hospitals may have better organization of care, and best-practice protocols [14].

Our study revealed some points that may be useful for policy intervention. Research organizations and payers could sponsor clinical quality improvement research to identify the care and treatment strategy differences among low-, medium-, and high-volume providers. Treatment strategies of the high-volume surgeons should be analyzed and utilized more widely in the country in order to decrease hospitalization costs and follow-up expenditure. For high-volume surgeons, payers may encourage them or consider additional reimbursement for them to serve as expert consultants to low-volume surgeons in order to improve healthcare quality and save costs. The organization of care system, practice protocols of high-volume hospitals may be further explored.

Our study has several limitations. First, the diagnosis of oral cancer, and any other co-morbid conditions are completely dependent on ICD codes. Nonetheless, the National Health Insurance Bureau of Taiwan has randomly reviewed the charts and interviewed the patients in order to verify the accuracy of the diagnosis. Hospitals with outlier chargers or outlier practice may undergo an audit and subsequent heavy penalties when malpractice or discrepancies are found. Second, the medical illness of oral cancer patients was evaluated with the Charlson Comorbidity Index score alone. The stages of oral cancer, which may be associated with different surgical procedures, were not included in the database. Third, the details of expenditure such as procedure, tests, and imaging can’t be clearly estimated from the present dataset. Future work related to this field is necessary in order to explore the difference of medical evaluation behaviors of different caseload surgeons and hospitals. Fourth, 30-day readmission rate and ED visits were used a proxy of quality measurement in our series. More detailed outcomes, such as quality of life and flap success rate may be needed to be explored in the future. Fifth, our series explored the direct cost or expenditure derived from the NHIRD and indirect cost such as time lost from work, and support person time lost from work can’t be extracted from this dataset [26]. Further study with primary survey linking administrative data and exploring whether controlling healthcare expenditure by payers will result in change of caseload and outcomes may be initiated. However, given the robust magnitude and statistical significance of the effects in this study, these limitations are unlikely to compromise our results.

### Conclusions

After adjusting for physician, hospital, and patient characteristics, low-volume provider incurred significantly higher costs and more follow-up expenditure per patient than did other providers with higher volumes. Treatment strategies, organization of care system, and practice protocols adopted by high-volume providers should be further analyzed and utilized more widely.

## Supporting Information

Appendix S1 **Methods for defining the caseload category of surgeons.**
(DOC)

Appendix S2 **Expenditure and additional information for the oral cancer patients.**
(DOC)
