# Supplementary material for: Association between Provider Volume and Healthcare Expenditures of Patients with Oral Cancer in Taiwan: A Population-Based Study
Source: PLoS One. 2013 Jun 4;8(6):e65077. doi: 10.1371/journal.pone.0065077 (PMC3672134; doi:10.1371/journal.pone.0065077)
Supplement: Appendix S1 — Methods for defining the caseload category of surgeons. (DOC) [file pone.0065077.s001.doc]

**Appendix S1**

1. The caseload of each provider was calculated.

2. Sorting the provider’s order by caseload number

3. Defining the category of caseload was as the following steps

| Provider ID | Caseload | Cumulative case |
| --- | --- | --- |
| 1 | 1 | 1 |
| 2 | 1 | 2  Roughly  1/3 cases of  oral cancer patients |
| 3 | 2 | 4  Low volume |
| 4 | 2 | 6 |
| . |  |  |
| . |  |  |
| 154 | 8 | 411 |
| 155 | 9 |  |
| 156 |  | Roughly  1/3 cases of  oral cancer patients |
|  |  | Medium volume |
|  |  |  |
| 184 | 21 | 864 |
| 185 | 22 |  |
| 186 | 22 | Roughly  1/3 cases of  oral cancer patients |
| 187 | 23 | High volume |
| . | . |  |
| 195 | . |  |
| 196 | 96 | 1300 |
