# Supplementary material for: Association between Provider Volume and Healthcare Expenditures of Patients with Oral Cancer in Taiwan: A Population-Based Study
Source: PLoS One. 2013 Jun 4;8(6):e65077. doi: 10.1371/journal.pone.0065077 (PMC3672134; doi:10.1371/journal.pone.0065077)
Supplement: Appendix S2 — Expenditure and additional information for the oral cancer patients. (DOC) [file pone.0065077.s002.doc]

| **Appendix S2**. Expenditure and additional data for oral cancer patients (n=1300) | | | |
| --- | --- | --- | --- |
| Characteristic |  |  |  |
| Age, years (mean ±SD) | 52±10 | |  |
| Oncological surgery cost (mean ±SD) | 11080±4645 | |  |
| Length of stay for oncological surgery (mean ±SD) | 24±12 | |  |
| Number of OPD visits during 12 months (mean ±SD) | 36±21 | |  |
| Number of hospitalization during 12 months (mean ±SD) | 2±2 | |  |
| Length of stay during hospitalization during 12 months (mean ±SD) | 17±29 | |  |
| OPD visits cost during 12 months (mean ±SD) | 6080±5374 | |  |
| Hospitalization cost during 12 months (mean ±SD) | 4049±7071 | |  |
| Outpatient & hospitalization cost (mean ±SD) | 10129±9248 | |  |
| Emergency department visits during 12 months | 22.2% | |  |
| Readmission within 30 days | 2.2% | |  |
| * CCIS: Charlson index score; SES: Socioeconomic status. | | | |
